# Quorum protection, growth and survival

**DOI:** 10.15698/mic2015.02.188

**Published:** 2015-01-23

**Authors:** Ian G. Macreadie

**Affiliations:** 1 School of Applied Sciences, RMIT University, PO Box 71, Bundoora; Victoria 3083, Australia; Tel: +61 3 9925 6627; Fax: +61 3 9925 7100

**Keywords:** cell culture, cell survival, inocula effect, quorum protection, unculturable microbes

## Abstract

For the growth of a cell culture, one inoculates not with one cell but with a quorum of cells. This most often a requirement, not just a convenience, and most of us take this for granted without question. Here this observation is re-examined to understand why a quorum may be required to grow cells. The importance of quorums may be widespread in the aspects of microbiology they affect. It is very likely that quorums are connected with and have a large impact on the determination of Minimal Inhibitory Concentrations. It is also possible that low cell density may adversely affect cell survival, however, this is an area where even less is known. The need for a quorum might affect other aspects of microbial cell culture, cell isolation and cell preservation. Effects also extend to mammalian cell culture. Here I seek to review studies that have been documented and speculate on how the information might be utilized in the future.

## INTRODUCTION

Rasmussen *et al*. 1 stated: “People growing cells have always known that cultures having low cell densities had difficulties in ‘taking’”. Indeed, it is certain that first microbiologists used quorums of cells to start their cultures so the observation goes back more than a century. Prior to that, for millennia those using starter cultures in traditional food processing, relied on a certain critical biomass to start their next culture. Only in the past six decades have studies started to document the growth of microbial cells in a quorum and the lack of growth without one.

Many would regard the growth of microbial cells to be a very robust procedure. Usually a loopful of cells is added to culture medium and after overnight incubation a dense culture of cells is observed. However, there are many occasions where growth does not occur. For example, if too few cells are inoculated growth might not occur, and if there is an antimicrobial agent present there is even greater likelihood of growth not occurring.

It is likely that problems with culture and maintenance at low densities can include aspects of death and well as lack of growth. Most studies have focused on growth where outcomes are clear and measured relatively easily. Insights come from the culture of ciliates where the 1951 Kidder and Dewey’s guide on culturing of *Tetrahymena* stipulated no less than a 1% inoculum be used 2. Five decades later Rasmussen *et al*. 1 clearly showed the death of *Tetrahymena thermophila* when inoculated at 250 cells/ml but immediate and good growth when then inoculum was 10-fold higher. Most significantly, cells at low density could be made to grow like those at 2500 cells/ml by simply adding ‘conditioned media’ from a culture of cells at 50,000 cells/ml. This indicates that Tetrahymena release materials that confer protection of the population. In this example there is protection of viability since there is death at low density. However, there could also be protection of growth of low density. Exactly what these natural quorum protection materials are remains to be determined. Their characterization requires isolation, analysis and re-testing to positively identify the protective molecules.

The concept of quorum protection is distinct from the well known phenomenon of quorum sensing. Quorum sensing molecules are also released by various microbial cells and at a critical concentration they dictate the cessation of growth. Quorum sensing molecules include homoserine lactones and peptides and have been found in Gram negative and Gram positive bacteria (reviewed in 3), as well as in eukaryotic microbes such as *Candida albicans* where farnesol and tyrosol are major quorum sensing molecules (reviewed in 4). Quorum sensing molecules are a means of ensuring the survival of a high density population and in that sense they also involve protection. However, the quorum protection concept being introduced here involves low cell density, different sets of molecules, and they affect different cellular pathways.

## QUORUM PROTECTION MOLECULES FOR GROWTH

The search for biochemicals conferring quorum protection is usually very direct. Rather than identifying secreted protective compounds, the approach has been to validate additives. Shousboe and Rasmussen 5 determined that lipids and long chain alcohols can achieve the quorum protection of ciliates, that was observed with conditioned media. Interestingly, while these lipids and long chain alcohols are not required for normal growth, this study showed they have a novel role in triggering proliferation.

Similarly, Friis *et al*. 6 showed that diluting Wickerman’s minimal synthetic medium 1200 fold caused the growth of *Saccharomyces cerevisiae*, inoculated at 1000 cells/ml, to lag for several days but this lag was greatly reduced by adding 70 μm Ca^2+^. Calcium is well known to be involved as a signaling molecule in the cell cycle and it appears from this work that it acts to drive entry into the cell cycle.

## "THE INOCULUM EFFECT": ANTIBIOTIC SENSITIVITY AND CELL DENSITY

In the testing of antimicrobial drugs a number of factors affect drug sensitivity. For example, the entry of drugs into cells is affected by cellular efflux pumps and such pumps can limit the drug concentration within cells. Another factor, low cell density effect is well known and is usually referred to as "the inoculum effect". It is of particular concern because drug testing studies are designed to determine a minimal inhibitory concentration (MIC), and what is observed is that a population of cells at low density are more inhibited than a population of cells at high density. Some of the studies where inoculum effects have been noted are shown in Table 1. For example, MacIntyre and Galgiani 7 showed the MICs of fluconazole and a test triazole increased dramatically when inocula densities were increased from 10^2^ cells/ml to 10^5^ cells/ml. Increases for fluconazole ranged from 4 fold to > 256 fold. Strains employed included *C. albicans*, *C. glabrata*, *C. lusitaniae* and *C. tropicalis*. In contrast, this is not applicable to all drugs. For example in their earlier study they saw no such effect when the inhibitory drug was amphotericin 8.

The inoculum effect has been attributed to a variety of causes. For example, sulfa drugs (analogs of *p*-amino benzoate (pABA)) are affected by cell density and by exogenous pABA, and often cited in textbooks as an example of a competitive inhibitor. It is considered that naturally occurring pABA released from dead cells may be the cause of the inoculum effect 9, however, quorum protection could be another explanation. To test this idea it would be of interest to determine the levels of pABA released by cells.

Steels *et al*. 10 found an "inocula effect" with the food spoilage yeast *Zygosaccharomyces bailii* in the presence of the food preservative sorbic acid. Their conclusion from the study was that the majority of cells were sensitive to sorbic acid but some cells in the population were phenotypically resistant. Upon sub-culture they behaved just like the parental strain. In their paper they reported a high non-cultivable rate of single cells; 1.3 survivors per 5 cells. Their work leaves open the possibility that yeast extract, in their growth medium, contains a quorum protective compound.

Is the inoculum effect related to quorum protection as well? Could quorum protection molecules have a role in survival under adverse circumstances (in the presence of antibiotics) as well as in normal growth media? There is a need to perform experiments to validate this. The use of "conditioned medium" might restore growth to cells at low density, removing the inoculum effect.

## STUDIES OF SURVIVAL

In our recent study in yeast we examined survival of *Candida glabrata* cells in water, without any other additives 11. Such conditions are also hypo-osmotic, adding another stress. When cells were suspended in water at low cell density they commenced dying exhibiting signs of apoptosis, including membrane flipping; within 5 days all cells had died. However, if the cell density was greater than 10^5^ cells per ml the yeast survived for at least 5 days. Granot and Snyder 12 showed no loss of viability of *S. cerevisiae* cells at 10^7^ cells/ml in water after 22 days. Clearly, cells at high density survive for a very long time, perhaps indefinitely.

Our study further showed that the yeast cells released biochemicals into the water that enabled survival. These "quorum protection" compounds were found to be released from cells and at < 10 mg/litre they could be applied to cells at low density to confer their survival. The compounds were less than 10 kDa in size as indicated by dialysis experiments. Current studies are on-going to determine the nature of the molecules necessary for quorum protection.

**Table 1:** Main features of *N. crassa* alternative NAD(P)H dehydrogenases.

**Observation**	**Given explanation**	**Reference**
Miconozole and 5 fluorocytosine inhibit clinical isolates of yeast more at low cell density	The physiology of microbes is more varied at higher density, affecting resistance	8
Inhibition of *S. cerevisiae* growth by sulfa drugs is more pronounced at low cell density	Dead cells provide pABA that competes with sulfa drug	9
*Zygosaccharomyces bailii* was inhibited more greatly by sorbic acid at low cell density	There were more phenotypically resistant mutants at high density	10

## FAILURE TO PROPAGATE ENVIRONMENTAL SAMPLES

The failure to culture cells at low density is a frustration that can often be remedied by ensuring that larger culture inoculums are made. However, for cells where stocks have not been established there may be ramifications such as the total inability to culture cells. This is a special concern for environmental samples.

The culture of microbial cells from natural sources is most frequently impossible. This observation, widely known as the "great plate count anomaly" refers to the inability of 99% of cells in soil to be cultured 13. Reasons for this phenomenon can often only be speculated. For example, the non-cultivable cells may be dead or they may have extremely long generation times, however, their presence can be demonstrated by DNA amplification, or by capture on filters followed by fluorescent staining of their DNA. Davis *et al.* 14 improved culture rates of soil bacteria with improved media, altered inocula and increased incubation times, however, most microbes remained uncultured. Another reason for the failure to culture soil samples may be due to the major differences in the laboratory environment, including the usual practice of transferring microbial cells to a medium that is liquid and rich in nutrients. For example, the culture of the bioleaching microbe *Acidithiobacillis ferridoxans* requires very low levels of nutrients and extremely acidic medium.

Another reason for failure to grow could be due to the low levels of quorum protection molecules. That is, while the total numbers may be high, the concentration of "like" microbes may be very low. If such microbes release quorum protection substances these will be in low levels, due to the low abundance of particular microbes.

## THE CONCEPT OF QUORUM PROTECTION

It has been known for a considerable time that the growth and survival of cells depends on communication between cells. Raff 15 reviewed this phenomenon in mammalian cells, concluding that for many mammalian cells death is the normal behaviour and that cells released survival signals that prevented death. Some of the molecules that confer survival to particular cells include testosterone, adrenocorticotrophic hormone, colony stimulating factors, fibroblast growth factor, interleukin-2. It is of interest that the survival molecules mentioned above are produced by heterologous cells: that is, cells from a certain tissue may produce the protective molecules for a different tissue. Indeed it is expected that advances in the survival of normal, non-transformed human cells could lead to major advances in regenerative medicine, since many of the current limitations relate to cell growth and death.

Some microbes also secrete biochemicals to protect "like neighbours" and the consequence of a low cell density is a lack of protection, through chemical signals, from "like" microbes. Therefore, this can equate to the second reason for the failure to culture. If we could add quorum protective compounds to the medium, the culture may be enabled. For example, Nichols *et al.* 16 found the pentapeptide LQPEV enabled the growth of an otherwise uncultivable *Psychrobacter* sp. strain on a standard medium.

## A QUORUM PROTECTION HYPOTHESIS

**Figure 1:**
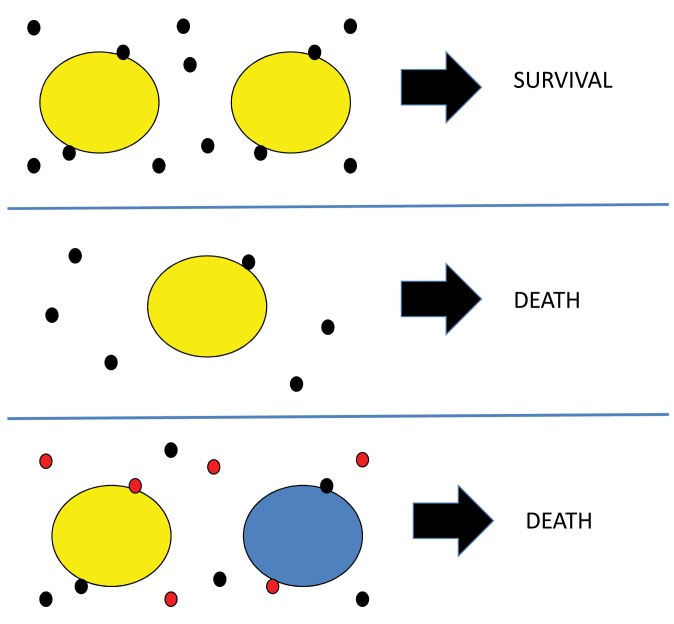
FIGURE 1: Some hypothetical scenarios in quorum protection. **Top panel:** depicts high cell density with cells being protected by 2 quorum protection molecules. Cells survive. **Middle panel:** depicts a cell with a low concentration of quorum protection molecules such that the threshold for survival is not reached. The cell dies. ** Bottom panel:** depicts a high density cell population that is mixed. Each cell produces specific molecules to protect their own species, but each can bind non-specific molecules. The low density of specific quorum protection molecules does not meet the threshold required for survival.

Figure 1 depicts my working hypothesis on quorum protection. In this example the threshold for quorum protection of cells is two specific molecules to enable cells to survive (Figure 1, top panel). At the lower cell density (Figure 1, middle panel), that threshold is not reached and cells die. The scenario shown in the bottom panel relates to mixed cultures, such as soil microbes where there may be a high density and high diversity of cells. If each cell is dependent on quorum protection molecules from "like" microbes the critical threshold for survival of the yellow and the blue microbes may not be reached, and both would die. Indeed, it is noted that around 99% of the microbes present do not grow under regular conditions used for isolation.

## INHIBITORS OF, AND INTERFERENCE WITH QUORUM PROTECTION

**Figure 2:**
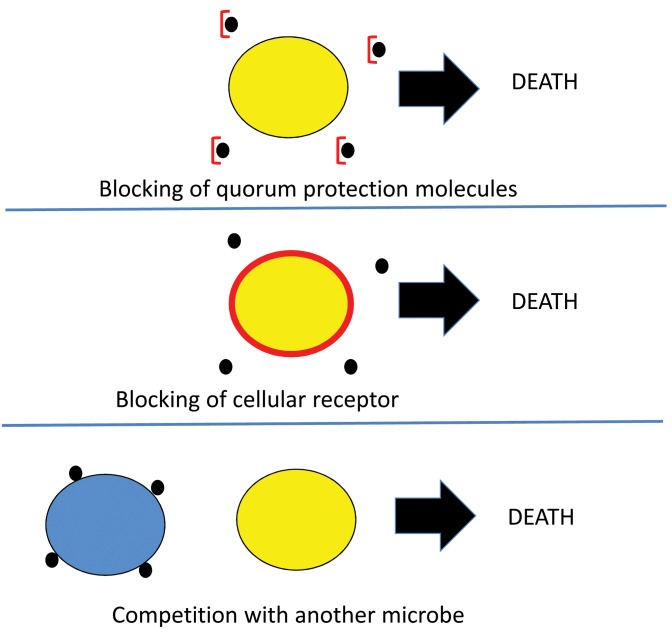
FIGURE 2: Some hypothetical scenarios on interference with quorum protection. **Top panel:** depicts molecules (e.g. EDTA) that bind the quorum protection molecule, rendering them ineffective in quorum protection. **Middle panel:** depicts blocking of the cellular receptor of quorum protection molecules. ** Bottom panel:**depicts a blue microbe competing for and depleting the quorum protection molecules needed by the yellow microbe.

Could quorum protection open up strategies for novel antimicrobial targets? As more knowledge is gained about the whole phenomenon about quorum protection molecules and their receptors, there could be many opportunities for new antimicrobial strategies. For example, one previously mentioned quorum protection molecule, calcium, can be chelated by EDTA and indeed EDTA is known to have antimicrobial activity. EDTA inhibits the ability of *Candida albicans* 17 and *Cryptococcus neoformans* to form biofilms 18. This scenario of inhibiting quorum protection by direct binding to a quorum protection molecule is depicted in Figure 2 (top panel). Similarly it could be hypothesized that the receptors for quorum protection molecules could also be blocked (Figure 2, middle panel), or that other microbes may compete for more and more efficiently sequester quorum protection molecules, leading to insufficient amounts for the dependent microbe, resulting in death of the yellow microbe (Figure 2, bottom panel). The blue microbe could survive if it can use the heterologous quorum protection molecules.

## CONCLUSIONS

Quorum protection is a new phenomenon in biology. It encompasses the new ideas that molecules are secreted to enable growth and survival. It remains to be determined what these molecules are, whether or not the same molecules serve both purposes, and how generic or specific they might be. Currently quorum protection appears to be a broad phenomenon with it appearing in yeast, *Tetrahymena* and mammalian cells, but will it be a universal phenomenon? If not, how might cells become resistant to survive without such protection? One could imagine that there must be strong selective pressure for microbes that do not require quorum protection. In the lab it may be relatively easy to select for resistant microbes, those that survive hypo osmotic stress. For example, an experiment to screen for a spontaneous resistant mutant in 10^7^ cells at 10^4^ cells/ml would require handling a litre of low density cells.

In mammalian cell culture, particularly culture of stem cells, the understanding of quorum protection may possibly provide opportunities for advances in cell culture. Finally, if we can find quorum protection molecules for non-culturable microbes then they will finally become culturable. This could lead to a revolution in industrial microbiology.
